# Influence of pH control in the formation of inclusion bodies during production of recombinant sphingomyelinase-D in *Escherichia coli*

**DOI:** 10.1186/s12934-014-0137-9

**Published:** 2014-09-12

**Authors:** Andrea Castellanos-Mendoza, Ricardo M Castro-Acosta, Alejandro Olvera, Guadalupe Zavala, Miguel Mendoza-Vera, Enrique García-Hernández, Alejandro Alagón, Mauricio A Trujillo-Roldán, Norma A Valdez-Cruz

**Affiliations:** Departamento de Biología Molecular y Biotecnología, Instituto de Investigaciones Biomédicas, Universidad Nacional Autónoma de México, AP. 70228, México, Mexico D.F, CP. 04510 México; Departamento de Medicina Molecular y Bioprocesos, Instituto de Biotecnología, Universidad Nacional Autónoma de México, Cuernavaca, Mor., Mexico; Unidad de Microscopía. Instituto de Biotecnología, Universidad Nacional Autónoma de México, Cuernavaca, Mor., Mexico; Instituto de Química, Universidad Nacional Autónoma de México, Mexico D.F, Mexico

**Keywords:** Inclusion bodies, Culture conditions, pH, E. coli, β-structure, Sphingomyelinase-D, Recombinant proteins

## Abstract

**Background:**

Inclusion bodies (IBs) are aggregated proteins that form clusters when protein is overexpressed in heterologous expression systems. IBs have been considered as non-usable proteins, but recently they are being used as functional materials, catalytic particles, drug delivery agents, immunogenic structures, and as a raw material in recombinant therapeutic protein purification. However, few studies have been made to understand how culture conditions affect the protein aggregation and the physicochemical characteristics that lead them to cluster. The objective of our research was to understand how pH affects the physicochemical properties of IBs formed by the recombinant sphingomyelinase-D of tick expressed in *E. coli* BL21-Gold (DE3) by evaluating two pH culture strategies.

**Results:**

Uncontrolled pH culture conditions favored recombinant sphingomyelinase-D aggregation and IB formation. The IBs of sphingomyelinase-D produced under controlled pH at 7.5 and after 24 h were smaller (<500 nm) than those produced under uncontrolled pH conditions (>500 nm). Furthermore, the composition, conformation and β-structure formation of the aggregates were different. Under controlled pH conditions in comparison to uncontrolled conditions, the produced IBs presented higher resistance to denaturants and proteinase-K degradation, presented β-structure, but apparently as time passes the IBs become compacted and less sensitive to amyloid dye binding.

**Conclusions:**

The manipulation of the pH has an impact on IB formation and their physicochemical characteristics. Particularly, uncontrolled pH conditions favored the protein aggregation and sphingomyelinase-D IB formation. The evidence may lead to find methodologies for bioprocesses to obtain biomaterials with particular characteristics, extending the application possibilities of the inclusion bodies.

**Electronic supplementary material:**

The online version of this article (doi:10.1186/s12934-014-0137-9) contains supplementary material, which is available to authorized users.

## Introduction

Bacteria like *E. coli* have been a successful cellular model to produce useful recombinant proteins in modern biotechnology [1]. Nevertheless, when heterologous protein over-expression occurs, an inefficient folding could occur, which together with the shortage of chaperones may promote protein aggregation [2],[3]. Those aggregates are called inclusion bodies [4],[5], and can be formed in the cytoplasmic or periplasmic area [6],[7]. IBs are dynamic reservoirs that contain a large amount of recombinant protein, various host proteins like chaperones, among other components of the cytoplasm [4],[8]. IBs are highly hydrated dense particles of porous structure [9],[10], their surface varies from rough to smooth [6], and their size is normally in the range of 50 to 700 nm, having spherical, cylindrical or ellipsoidal teardrop shapes [10]-[15].

It has been demonstrated that inside an IB there are heterologous and host proteins combining native-like structures with partially folded and misfolded proteins [13],[16]-[21]. The IB formation and its maintenance involve a complex network of intracellular responses related to culture conditions, leading to complex and stable structures sometimes showing bioactivity [13],[22]. Due to their different physicochemical properties, IBs have been proposed for various uses, such as catalysts, support materials, drug delivery agents, cell therapy, and immunogens, and their recent application has become an important new topic in biology, medicine and biotechnology [11],[23]-[30]. However, the study of their physicochemical properties is a recent area, and few reports have been published about that [18],[31]-[33].

The IB characteristics, such as size, geometry, composition, and conformation, are associated with the host strain employed, culture conditions, and medium, and also the recombinant inducer used [6],[13],[25],[34],[35]. Margreiter et al. [35] reported that in fed cultures of *E. coli* K12 producer of β-lactamase, the size of the IBs increased over the cultivation time (an increase of 200 nm after 25 h). The IB size was also affected by the different concentrations of IPTG, decreasing with the decrease in IPTG concentration [34],[35]. Importantly, an analysis by sedimentation field-flow fractionation (sedFFF) determined that cultures with part-induction strategies resulted in broader IB size distributions and higher overall protein yields [34]. By using asymmetrical flow field-flow fractionation-multi-angle light scattering (AsFlFFF-MALS), IBs of green fluorescent protein were about 700 nm irrespectively of the induction times and IPTG concentrations in cultures at 30°C, but in cultures at 37°C the IB size is determined by the induction time [12]. Furthermore, it has been reported that culture time increases the resistance of IB to trypsin degradation [10], implying differences in protein conformation inside them. The culture time has also been associated to the IB shape, finding that early-culture-time IBs were spherical, and at the end of culture IBs were cylindrical or spherical [14].

Different culture strategies have been used to prevent protein aggregation, but scarce approaches are proposed to produce IBs with determined properties [27]. For example, a culture temperature decrease often improves protein solubility as well as decreases the IB size [36]-[40]. Meanwhile, the protein accumulation in IB is favored at temperatures above 37°C due to the augmentation in hydrophobic interactions and β-sheet contents [13],[41]-[43]. Thus IB become more stable to chemical denaturation and proteolysis when the temperature increases [16]. Nevertheless, the activity in aggregates inversely correlates to the temperature [16]. In thermoinducible systems, the IB formation has also been attributed to the increase in recombinant protein synthesis rate and mRNA overexpression [43], recombinant protein amount [42]-[46], and activation of some heat shock proteins that could favor the disorder in folding reactions [3],[42],[43],[47]. Furthermore, IB formation is favored by shake flask conditions using chemical [13] or thermo-inducible [48] recombinant strains. Importantly, it has been demonstrated that under uncontrolled pH conditions using a thermo-inducible strain cultured in shake flasks, the pH declined to 4.8 and caused an increase of IB formation [48]. Likewise, under uncontrolled conditions in bioreactors, IBs were also formed. However, it was described that under controlled pH conditions the IB aggregation decreased about 50% [48].

The tick *Boophilus microplus* (known also as the cattle tick or southern cattle tick) is an economically important parasite found in a variety of livestock species. This is globally distributed with an important presence in Asia, parts of Australia, Madagascar, Southeastern Africa, the Caribbean, South and Central America and Mexico [49],[50]. Tick saliva contains numerous molecules like sphingomyelinase-D (SMD) that might modulate host immune responses [51] and combined with other proteins has been proposed as blood meal strategy for the tick [52]. The SMD from tick *B. microplus* has a molecular weight of 33.1 kDa, a conserved structure of (α/β)8, and a theoretical pI of 6.04. Since small quantities of SMD are produced per tick, its recombinant production is biotechnologically important to determine the mechanisms involved in its participation in blood feeding, to develop new antisera against this protein and the possible future development of a control against tick infestations.

Nowadays, several bioprocesses accumulate high percentage of recombinant protein in IBs to facilitate large-scale recovery by centrifugation [53]-[55]. Hence, it is important to understand how culture conditions modify the aggregation properties inside IBs and how they maintain certain characteristics to extract active proteins or proteins with determined conformations. Therefore, in this work we studied the effect of uncontrolled pH cultures versus controlled physiological pH (7.5) cultures, on the physicochemical properties of the IBs produced in *E. coli,* which heterologously produce SMD from the saliva of tick *B. microplus,* as a protein model.

## Results

### Variations in pH affect the growth and recombinant protein production

**Figure 1:**
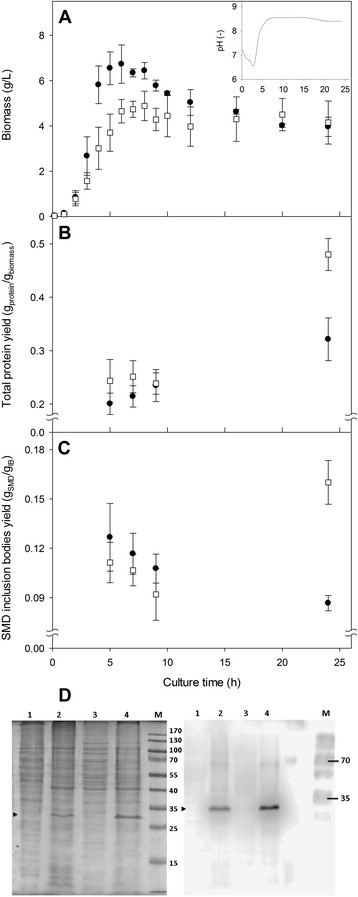
**Biomass growth and production of sphingomyelinase-D (rSMD) from tick (** ***Boophilus microplus*** **) by a recombinant strain of** ***E. coli*** **BL21-Gold (DE3). A**. Kinetics of biomass growth for the recombinant strain of *E. coli* BL21-Gold (DE3). In the inset, the evolution of pH on uncontrolled cultures is shown. Data show the average and the standard deviation of the 24 h cultures that were carried out by quadruplicated. Cultures were carried out under controlled pH at 7.5 (open squares), and uncontrolled pH (closed circles). **B**. Kinetic behavior of the total protein yield based on biomass dry weight, after chemical induction. Data shows the average and the standard error of duplicate determination of total protein and biomass. **C**. Kinetic behavior of rSMD yield based on total protein in inclusion bodies, after chemical induction. Data show the average and the standard error from two samples recovered from independent cultures (5, 7 and 9 hours of culture). The average and standard deviation from quadruplicate cultures is shown at 24 h of culture. **D**. Comparative SDS-PAGE (left) and Western blot (right) of cytoplasmic soluble proteins and those obtained from solubilized IB (with 10% of SDS), obtained at 20 h post-induction (24 h of culture). Lanes 1 and 2, soluble and IB proteins harvested from uncontrolled pH cultures. Lanes 3 and 4, soluble and IB proteins from controlled pH cultures. M means molecular weight marker standard.

The effects of the pH variation in cultures carried out at a controlled pH (7.5 ± 0.1) and uncontrolled pH on biomass growth, total protein and rSMD yields were evaluated (Figure 1). By controlling the external pH at 7.5 ± 0.1, the cytoplasmic pH is maintained in the same range simulating the physiological *E. coli* conditions [56]. In all cultures, the dissolved oxygen tension was controlled at 30% (with respect to air saturation) in order to avoid oxygen limitations and organic acids overproduction [57],[58]. Glucose was consumed at the same rates by controlled and uncontrolled pH cultures, and a small concentration of lactic acid was detected (<0.3 g/L) in both pH strategies (data not shown). At uncontrolled pH, the maximum biomass concentration achieved was approximately 28% higher than at controlled pH condition, at the end of the exponential growth phase (Figure 1A). However, in both cultures a similar biomass was obtained at the end (24 h) of cultures (4.2 ± 0.9 g/L). No lag phase was found in both cultures, but significant differences were observed in the specific growth rate: 1.34 ± 0.06 h^-1^ in uncontrolled pH cultures, and 1.21 ± 0.04 h^-1^ in controlled pH conditions. In the inset of Figure 1A a typical profile of the pH for said cultures under uncontrolled pH is presented. Initially, the pH lowered down to 6.5 within the first 3 h, then increased up to 8.5 after 6 h of culture, and remained in this condition throughout the culture. The chemical induction in all cultures was made at 4 h, under uncontrolled pH cultures the induction occurred when the pH was near 7.4 (Figure 1A).

The influence of the pH culture strategy over the total protein yield on biomass (Y_prot/biom_) is shown in Figure 1B. After induction, Y_prot/biom_ was similar for both culture conditions, but after 24 h almost 50% more protein was obtained under controlled pH (0.48 ± 0.03 g_prot_/g_biom_) than at uncontrolled pH (0.32 ± 0.04 g_prot_/g_biom_). The yield behavior of the rSMD in IB (g_SMD_/g_IB_), quantified by densitometry on gels stained with Coomassie Blue is shown in Figure 1C. An rSMD enrichment in the IB was obtained (0.16 ± 0.01 g_SMD_/g_IB_) at the end of pH controlled cultures, while a small decrease of this yield was observed in uncontrolled cultures (0.09 ± 0.01 g_SMD_/g_IB_). The final differences in rSMD yield in IB can be seen on SDS-PAGE and Western Blots (Figure 1D), between controlled cultures (lane 4), and uncontrolled cultures (lane 2). This data demonstrated that rSMD accumulation occurs preferentially at controlled pH conditions (Figure 1C and 1D). In addition, a reduced amount of rSMD in the cytoplasmic soluble fraction was detected at controlled pH cultures (lane 3). At uncontrolled pH cultures no soluble rSMD was observed (lane 1, Figure 1D).

### Effects of controlled-pH and uncontrolled-pH strategies on IB size and morphology

**Figure 2:**
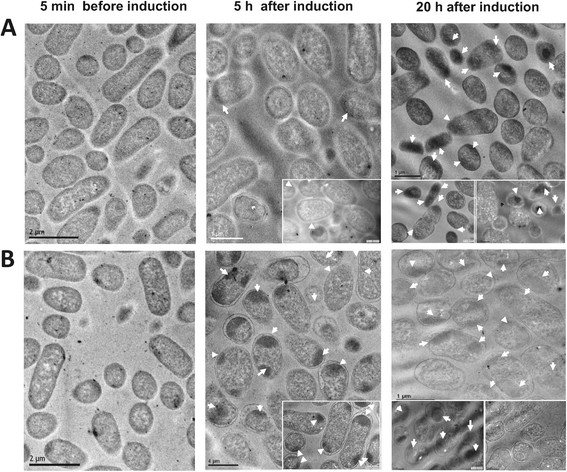
**Cross-sections of** ***E. coli*** **bacteria producing rSMD viewed under the transmission electron microscope (TEM). A**. Examination of *E. coli* BL21-Gold (DE3) cells cultured under controlled pH 7.5. **B**. TEM micrographs of *E. coli* BL21-Gold (DE3) cells cultured under uncontrolled pH. Cells harvested at non-induced time (left) (scale bars 2.0 μm), 5 h post-induction (middle), and 20 h post-induction (right) (scale bars of 0.5 and 1.0 μm). Inclusion bodies are marked with arrowheads.

The effect of the pH on the IB morphology inside cells and their size were visualized by transmission electronic microscopy (TEM) on fixed cells (Figure 2), as has been performed by others [13],[59]. Cells were harvested 5 min before induction, and 5 h or 20 h after induction (induction was performed at 4 h). The micrographs show cross-sections of *E. coli* producing rSMD. Under uncontrolled pH condition the formation of aggregates was favored (Figure 2). After 5 h of induction, around 61% of cells with at least one IB were observed in uncontrolled pH cultures, whereas at controlled pH only 7% of cells presented one (or more) IB.

After 20 h of induction, IB formation was observed in both culture strategies; at uncontrolled pH conditions, around 58% of cells presented at least one IB, in contrast with controlled pH where 31% of the cells contained one or more IB (Figure 2). Moreover, the IBs formed at uncontrolled pH conditions were larger; almost 65% of the IBs observed were ≥ 500 nm. While at controlled pH, 65% of the IBs presented sizes smaller than 500 nm.

**Figure 3:**
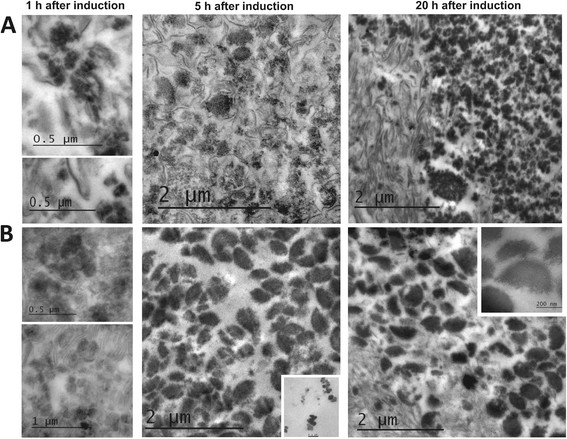
**Electron micrographs of purified rSMD IB. A**. IBs from cultures under controlled pH 7.5. **B**. IBs purified from uncontrolled pH cultures strategies. 1 h post-induction (left) (scale bars of 0.5 and 1.0 μm), 5 h post-induction (middle) (scale bars 2.0 μm), and 20 h post-induction (right) (scale bars 2.0 μm). Small IBs found at 5 h post-induction (**B** middle inset) (scale bar represents 0.1 μm). Examination of IBs produced after 20 h of induction (**B** right inset) (scale bar represents 200 nm).

Said differences were also observed with the analysis of purified IBs by TEM at three culture times in both pH strategies (Figure 3). Micrographs indicated that IB formation in both cultures occurred at least during the first hour after induction. During the first 5 h, the aggregation was favored by forming preferentially larger IBs at uncontrolled pH conditions, compared to smaller IBs at controlled pH conditions, although at uncontrolled pH small IBs were also observed (<100 nm), as is shown in the inset in Figure 3B at 5 h. Nevertheless, at controlled conditions large aggregates seem to be formed by joined small IBs (Figure 3A). Differences in IB size also were evident after 20 h of induction; being larger in size the IBs from uncontrolled pH cultures compared to those formed at controlled pH conditions. The inset of Figure 3B (at 20 h) shows IBs of about 450 nm in diameter (no pH control). It should be noted that these IBs were recovered in the same buffer (pH 8).

**Figure 4:**
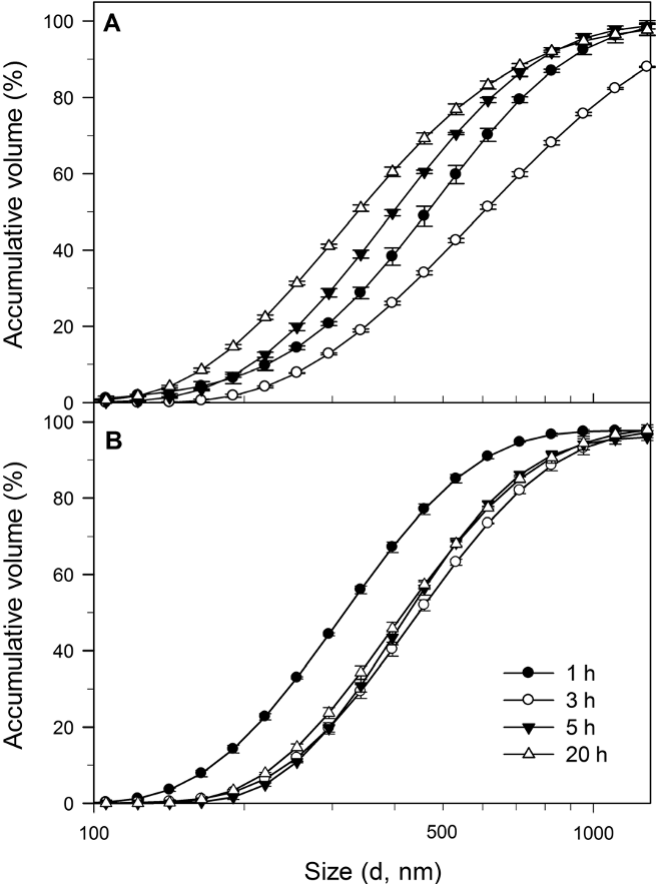
**Comparison of logarithmic cumulative volume (%) distributions of the rSMD IB sizes harvested at 1, 3, 5 and 20 h after induction under controlled pH 7.5 condition (A), and under uncontrolled pH strategy (B).** Distribution of IB size was determined in a Particle Sizer (Zetasizer Nano). The graphs were cut at 1300 μm because the contribution to scattering by particles > 1300 μm in size was only approximately 3%, except in data from controlled pH cultures at 3 h post-induction (12%). Figures show the accumulated values ± standard deviation of data from experiments performed by triplicate.

In order to facilitate the understanding of the results and their discussion, logarithmic cumulative distributions [60] of the IB hydrodynamic diameters are presented in Figure 4. The IB size of all samples follows a unimodal log-normal distribution, as previously reported [12],[61]. This IB size distribution of samples collected at four cultures times (1, 3, 5 and 20 h after induction) from two pH culture strategies, was measured in a Particle Sizer (Zetasizer Nano, Malvern Inst. UK, Figure 4). The comparability was based on the maximum size reached by 50% of the IB population (IB_50_), and the mean/IB_50_ ratio (as described in Materials and methods).

In controlled pH cultures, the IB_50_ and the mean were 458 and 800 nm in diameter, respectively by the first post-induction hour. These sizes increased up to 615 and 1112 nm after 3 h post-induction (IB_50_ and mean, respectively). Then, IB_50_ and mean decreased after 5 h (396 and 735 nm) and 20 h (341 and 694 nm) post-induction. In uncontrolled pH cultures the IB_50_ and the mean increased from 1 h after induction (320 and 659 nm, respectively), to 3 h (458 and 802 nm), and remained similar from 5 h (432 and 740 nm) to 20 h (430 and 780 nm). Moreover, to have an idea of the broadness and the deviation of the log-normal distribution of IB sizes, the polydispersity of data calculated as the IB_50_/mean was in the range of 1.6 to 2.0 in all samples.

### Resistance of IB to proteinase-K degradation and its solubilization in guanidine hydrochloride

**Figure 5:**
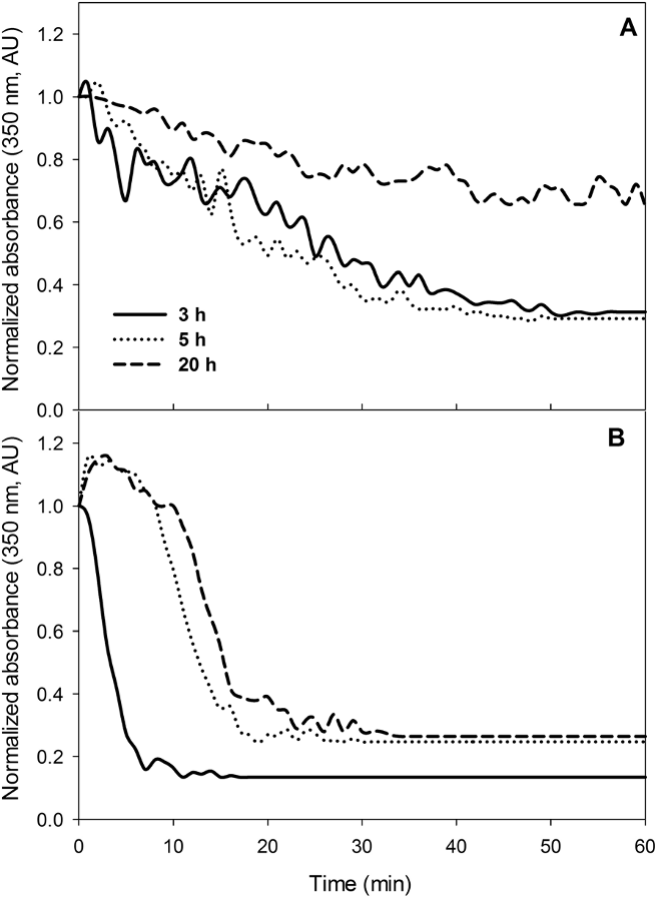
**Kinetics comparison of proteinase-K digestion of rSMD IBs harvested at 3, 5 and 20 h post-induction. A**. Digestion of IBs collected under controlled pH. **B**. Digestion of IBs obtained from uncontrolled pH strategies. The progressive degradation was followed by absorbance and data were normalized. Data show the average of triplicate experiments.

To understand the effect of pH variations on the aggregation and physical properties of IBs, the kinetics of differential disintegration by proteinase-K [61],[62] on purified IBs recovered at different culture post-induction times are presented in Figure 5. All experiments start with the same protein content (150 μg/mL), and a normalization of the absorbance was done. The IBs formed under constant pH 7.5 at 3, 5 and 20 h were found to be resistant to proteinase-K since only 60, 55 and 20% of disintegration, respectively, was observed after 30 min of incubation (Figure 5A). Whereas, a rapidly disintegration was observed with IBs harvested at 3 h post-induction under uncontrolled pH conditions. The end of the proteinase-K reaction in IBs collected at 3, 5 and 20 h was reached after 7, 18 and 23 min, respectively (Figure 5B).

**Figure 6:**
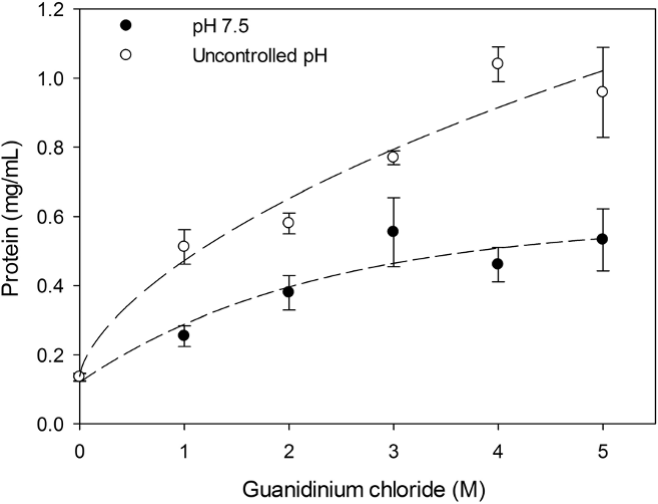
**Solubilization profiles of purified rSMD IBs collected at final culture time using different concentrations of guanidinium chloride.** Solubilization of rSMD IBs collected from controlled pH conditions (filled cirles) and IBs harvested in uncontrolled pH conditions (open circles). Graphs present the protein solubilization quantified by 2D quant method and each experiment was performed by triplicate.

The solubilization of IBs collected at the end of cultures using different concentrations of guanidine hydrochloride [63] was analyzed (Figure 6). The denaturation profiles showed significant differences from the addition of 1.0 M of GnCl agent. Almost a complete solubilization of the IBs recovered from uncontrolled pH cultures occurred at 4.0 M of GnCl, whereas the IBs from controlled pH cultures reached 40% of solubilization at 5.0 M of GnCl (Figure 6). Therefore, the IBs formed under controlled conditions presented an improved resistance to GnCl and proteinase-K degradation.

### Dye binding to structures in IBs produced under different pH conditions

The conformation of proteins inside an IB has been determined by binding to Congo red (CR), which recognizes entities enriched in β-pleated fibrillar conformation [64]-[66], and to Thioflavin-T (Th-T) which has been described as a dye that binds to the β-sheet surface along channels structured by "Cross-strand ladders" [67]-[69]. Hence we determined the CR and Th-T binding properties of IBs obtained at different culture times.

**Figure 7:**
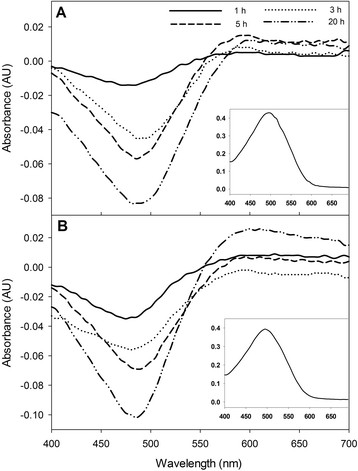
**Differential spectra of Congo-Red (CR) binding to rSMD IBs obtained from controlled (A) and uncontrolled pH cultures (B).** The rSMD IBs were isolated at 1, 3, 5 and 20 h post-induction. CR spectra were obtained in the presence of IB showing the absorbance shift at ~575 nm. In insets, the spectra for Congo red alone are shown.

The spectrum for CR alone exhibited a maximum absorbance at 490 nm [61],[65] as it is shown in the insets of Figure 7. When CR binds to amyloid material in the IB, the signal shifts to higher wavelength from 550 to 565 nm. The comparison of the absorption spectra shows differences between the IB produced under controlled and uncontrolled pH conditions. As time increases, an increase in the IB binding to CR was observed at uncontrolled conditions (Figure 7B) showing broad peaks at 565 nm. Whereas, IBs from controlled pH cultures show a small shift of absorbance (Figure 7A), indicating that both types of IBs had an amyloidogenic nature. Two CR binding experiments were carried out for each culture time from two different cultures.

**Figure 8:**
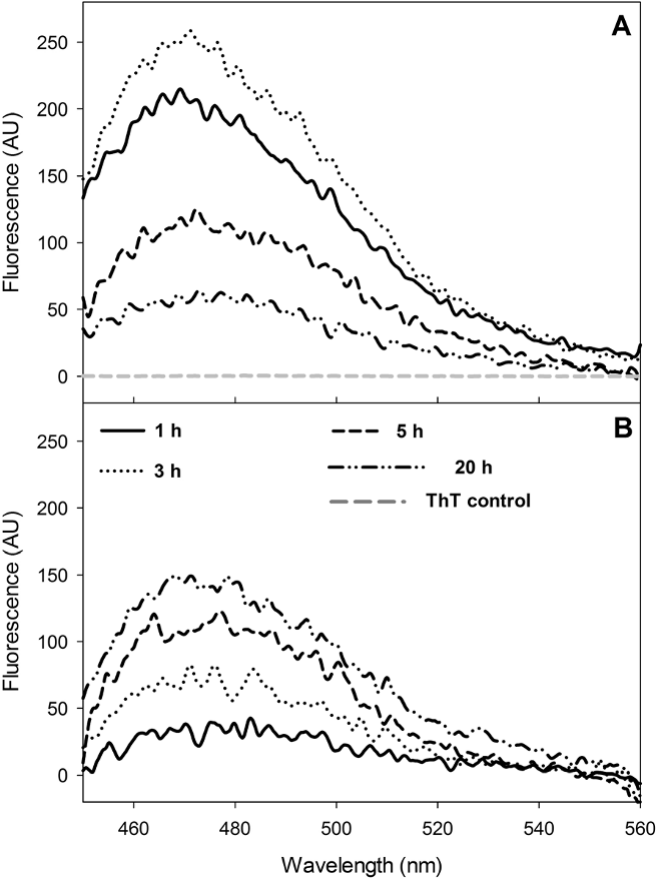
**Emission spectral characteristics of Th-T binding with rSMD IBs harvested at 1, 3, 5 and 20 h post induction. A**. Th-T spectra with IBs collected under controlled pH conditions. **B**. Th-T spectra with IBs recovered from uncontrolled pH cultures. An emission spectrum of Th-T alone is shown in A as a grey lane near zero. Concentrations of Th-T and IBs used for assay were 75 MM and 50 μg/mL respectively.

The intercalation of Th-T into extended β-sheet of amyloid structures in IB was measured as the enhancement of the maximum fluorescence emission compared to free Th-T dye [70]. The fluorescence spectra of Th-T incubated with IBs harvested at different times of culture are compared in Figure 8. The maximum fluorescence emission was around 465 and 475 nm for controlled and uncontrolled conditions. It can be observed that IBs formed under uncontrolled pH conditions and recovered 1 h after induction, exhibited limited binding to Th-T, and to CR. Furthermore, the fluorescence emission increased with time, reaching a maximum intensity of 150 AU at the end of culture (Figure 8B). Nevertheless, IBs aggregated at constant pH conditions, formed at 1 h and 3 h after induction, presented a maximum of fluorescence intensity of 214 and 258 AU, respectively. Finally, the fluorescence decreased in IBs harvested after 5 h and 20 h of culture (Figure 8A).

## Discussion

The formation of IBs and the nature of intermediates involved in aggregation, are determined by the biochemical properties of the proteins [9],[61],[71] and the environmental production conditions [16],[35],[72]-[74]. The IB formation is enhanced in uncontrolled pH strategies, compared to controlled pH [48]. Furthermore, it has been reported that the pH affects the tendencies of β-peptides to form amyloid deposits *in vitro*[75] that display similar features with IBs [9]. Then, the cytoplasmic pH conditions in *E. coli* will be crucial in the IB formation, their secondary structural determinations and their properties. Here, we presented our main findings relative to how controlled cultures at physiological pH (7.5) or uncontrolled pH conditions affect the sphingomyelinase-D inclusion bodies formation and their physical-chemical properties. We observed protein aggregation under uncontrolled pH culture conditions, similar to experiments reported where SpA-β-galactosidase was expressed under thermo inducible promoter culturing *E. coli* RR1 in shake flask or bioreactor [48],[76]. In those cultures, the pH declined from 6.7 to 5.1, but in our results the pH was initially acidified from 7.4 to 6.8 (inset of Figure 1), and then increased up to 8.5 (induction was made at pH 7.4).

To analyze the IBs size during cultures inside cells and purified IBs we used TEM, which was complemented with DLS, considering that DLS only provides an insight to the hydrodynamic diameter of the IB and not in its form [12],[23],[25],[61], *i.e.* from spheres to ellipsoids up to the rod like shapes (Figures 2 and 3). Under controlled physiological pH conditions (7.5), the rSMD aggregation to form IBs was slow and their sizes were smaller compared to those formed under uncontrolled pH conditions (Figures 2, 3, and 4). Moreover, at constant pH, active rSMD in a soluble form was obtained (Figure 1D), which was detected qualitatively (data not show) by the assay of sphingomyelinase activity [77]. It is important to note that although the percentage of rSMD in inclusion bodies was about 8 to 16% in the two conditions, the nucleation was sufficient to cause the formation of IBs, as has been observed by others [48]. The aggregation in large IBs at uncontrolled pH conditions was favored with culture time (Figures 2B, 3B, and 4B), compared to those IBs formed under at controlled pH where by the first hour of post-induction small aggregates were observed, as well as at the end of the culture (Figures 2A, 3A and 4A). This suggests that under controlled pH conditions, the entry to the stationary phase might have caused shrinkage of the IB relative to time. In Figure 3A, IBs appear to be more compact than those obtained in uncontrolled pH cultures. The presence of disordered fibers (observed mainly in controlled pH) could be due to globular protein elements forming complex macro-aggregates. Similar fibers structures have been observed previously [78],[79]. In addition, the differences in IBs size probably are due to differences in nucleation and IB growth properties during formation, as well the host proteins that interact with them. Differences in the composition of IBs were observed in SDS-PAGE at the end of cultures (Figure 1D). Then, in order to understand the effect of pH on the IB composition and on the host proteins involved in IB formation, it would be interesting to perform a proteomic approach during the post-induction processes.

The heterologous expression of sphingomyelinase-D in *E. coli* under uncontrolled conditions resulted in larger IBs, with more available protein extractable by proteinase-K (Figure 5B), compared to the resistant nature of those IBs produced under controlled pH conditions. Anyway, the resistance to proteinase-K activity of the IBs produced with any of the culture strategies increased over time. Data obtained from proteinase-K digestion were in agreement with those obtained from solubilization with guanidinium chloride (Figure 6). It has been described that proteinase-K selectively cleave the peptide bond adjacent to the carboxyl group of aliphatic and aromatic amino acids located in hydrophilic domains as loops and α-helical, like non-infectious cellular prion protein [80],[81]. Therefore, peptide bonds located inside or close to β-strands are partially resistant to proteolysis [82],[83], as prion proteins composed preferentially by β-sheet [62],[83]. Then, this suggests that IBs formed under constant pH presented more β-sheet conformation. Likewise, IBs produced at physiological (controlled) and uncontrolled pH conditions have differences in aggregation and structure composition.

Furthermore, the IBs produced under the two pH culture strategies, showed binding to amyloid specific dyes Th-T and CR. Particularly, the IBs formed at uncontrolled pH conditions presented binding to both dyes, which increased with time. This indicates that the amyloidogenic characteristic of those IBs, in conjunction with α-helix structures, β-extended conformation and random coils, allow their rapid disintegration by proteinase-K. Thus, the pH variation might activate the expression of proteins related to the pH stress and homeostasis [84], contributing to the different protein compositions of the IBs in comparison to those produced at controlled pH (Figure 1D, lane 2 and 4). In contrast, IBs formed under controlled pH conditions presented low CR interaction but showed high binding to Th-T at the initial times. After 5 h post induction the affinity for Th-T decreased. This may indicate that the interaction of Th-T along the channels of β-sheet surfaces was diminished [9], probably due to the IB size reduction observed by light scattering after 5 h post induction (Figure 4A), or because of a conformational change that limited the Th-T interactions. Overall, differences in CR and Th-T binding to the IBs could be due to the variation of the aggregation at the beginning of the IB formation, the secondary structural elements present inside aggregates, and the arrangement of proteins and their proportion inside the IBs.

It has been described that "non-classical" inclusion bodies are soluble in mild denaturants concentration, susceptible to degradation by protease and less amyloid nature [14],[22],[71]. Interestingly, the IBs produced under uncontrolled pH conditions presented a "non-classic" nature, being less resistant to degradation by proteinase K and GnCl, and presented less binding to Th-T, compared to IBs formed under controlled conditions, which were resistant to degradation by proteinase-K and GnCl, and exhibit higher affinity for Th-T. Hence, these last IBs were formed as "Classical inclusion bodies".

Different reports had demonstrated the recovery of "non-classical" IBs with recombinant protein in active form, produced in shake flasks under uncontrolled conditions, such as pH and dissolved oxygen [25],[61],[71], regardless of their secondary and tertiary structure. These results are in agreement with our results in uncontrolled cultures where the obtained IBs presented sphingomyelinase activity (data not show).

The cytoplasmic pH is normally maintained within a range of 7.4 to 7.8 when the external pH is in the range of 5.0 to 9.0 in suspension or 5.0 to 8.0 in adherent *E. coli* cultures [56],[85]-[88]. In adherent *E. coli* cultures the cytoplasmic pH is similar to the external pH of 8.5 to 9.0 [87]. Whereas, in suspended cells at pH 9.0, *E. coli* has an inverted pH differential between the external and the internal cell membrane becoming less alkaline [56],[86],[89],[90]. Moreover, in suspension cultures at high pH, proteomic data proposes that cells have strategies to reach the pH homeostasis, including acid production through amino acid catabolism using deaminases and sugar fermentation that presumably release acids, which neutralize alkalinity [91]-[95]. Also, it has been suggested the inward flow of protons through cation/proton antiporters, the proton capture via the F_1_F_0_-ATPase, and the reduction of cytoplasmic protons loss [56],[90],[96]-[98].

In addition, other experiments demonstrated that cell homeostasis responses [86]-[88], and the recombinant protein production process occur in a similar time order [99]. Then, we can hypothesize that during those cell responses, cellular microenvironments favor the nucleation and formation of proto-aggregates, which later allow the formation of IBs. The alkalization of the culture medium can cause small cytoplasmic changes forming environments that favor the accumulation and precipitation of host and recombinant proteins, whose isoelectric points are similar to the perturbed cytoplasmic pH. This study showed how the variation in pH due to *E. coli* metabolism during recombinant expression modified the formation of IBs beyond that aggregation directed by physical and structural characteristics of recombinant proteins. Furthermore, stress proteins such as those coded by *ibpB*, *lon*, *dnaJ*, *dnaK*, *clpB*, *clpX* and *grpE*, among others [97] might be involved in maintaining proteostasis (protein folding homeostasis), as a response to recombinant protein production and pH external changes.

## Conclusions

Results presented here demonstrate that under different pH conditions, the IB formation and their characteristics changed over the culture time. Particularly, under uncontrolled pH conditions, rSMD IBs formation was favored with non-classical IB characteristics, while those formed under controlled conditions were more resistant to proteinase-K degradation, a usual characteristic of classical IB. Information presented could be useful to reproducibly produce biomaterials with specific features, and to develop better protein recovery processes.

## Materials and methods

### Chemicals and reagents

Tris buffer, glycine, sodium dodecyl sulphate, phenylmethylsulfonyl fluoride (PMSF), and deoxy cholic acid were from Amresco (USA). Ammonium persulphate, acrylamide and bis-acrylamide, TEMED and EDTA, were from Biorad (USA). Coomassie Brilliant Blue R-250, Nonidet-P40, DNase I, Triton X-100, Bovine Serum Albumin (BSA), Congo red, IPTG, urea, proteinase-K, thioflavin-T, ampicillin and kanamycin were from Sigma-Aldrich (USA). SDS-PAGE molecular weight marker was purchased from Fermentas Thermo Scientific (USA). Glucose and NaCl from REASOL (Mexico), all other reagents were from J.T. Baker (USA). Paraformaldehyde, glutaraldehyde, osmium tetraoxide, Epon/Araldita, uranyl acetate and citrate were from Electron Microscopy Sciences (USA).

### Strain, plasmids and culture conditions

The coding gene for SMD from saliva of the tick *Boophilus microplus* (GeneBank KJ854238) was under the control of the phage T5 promoter in the expression plasmid pQE-30 (Qiagen, USA), and transformed in *E. coli* BL21-Gold (DE3) cells. A cryovial with 2.0 ML of recombinant *E. coli* (20% glycerol) with an optical density (OD 600 nm) of 1.5 AU (kindly provided by Dr. Alagón), was grown in two 250 ML Erlenmeyer flasks with 50 ML of culture media, at 37°C and 200 rpm overnight (C25I, New Brunswick - Eppendorf Co. USA), in the presence of ampicillin (50 μg/mL). All shake flasks and bioreactor cultures were grown on Super Broth medium (3.2% w/v peptone, 2% w/v yeast extract, and 0.5% w/v NaCl). Both shake flasks were cultured overnight and used to inoculate a 1.0 L bioreactor (Applikon, Netherlands) with an initial OD 600 nm of 0.1 AU (Spectronic Genesys 20, Thermo USA), where 1 OD was equivalent to 0.50 g dry cell weight per liter, similar to the data obtained by Baig et al. [4].

The batch cultures were carried out at 37°C with an operation volume of 600 ML. Dissolved oxygen tension (DOT) was controlled at 30% (with respect to air saturation) by cascade changing the agitation speed (between 200 and 900 rpm), and enriching the air with pure oxygen when required, maintaining an airflow of 0.6 L/min (1 vvm), by using a proportional-integral-derivative (PID) control strategy [100]. The culture medium was adjusted prior to inoculation to pH 7.5 in either of two conditions (controlled and uncontrolled pH conditions). In controlled cultures, pH was maintained at 7.5 by using an automatic addition of NaOH (1 M) through an on-off control strategy. In uncontrolled cultures, the pH varied freely according to the cell metabolism. Foaming was controlled by addition of silicone based antifoaming agent (Corning®, USA), when required. DOT, temperature, agitation, and pH were controlled by ADI-1030 and/or ADI-1010 Biocontrollers (Applikon, Netherlands), displayed online and stored in a hard drive for further analysis using the BioXpert® data acquisition program (Applikon, Netherlands). The chemical inductor, isopropyl-β-D-thiogalactoside (IPTG), was added before the pre-stationary phase at a final concentration of 0.1 MM. Glucose and lactate were measured using YSI2900 (YSI Life Sciences, USA). The data presented in this manuscript show the average and the standard deviation of the 24 h cultures that were carried out by quadruplicated.

### Total, soluble and IB protein separation and quantification

The biomass was recovered by centrifugation at 7000 × g for 10 min, at each sampling time. The cell pellet was suspended in 50 MM TrisHCl, 100 MM NaCl, 1 MM of EDTA and 1 MM of PMSF. The cell suspension was sonicated in a SoniPrep150 (Sanyo-Gallen-Kamp, UK) with an amplitude of 10 microns in 10 steps of 30 s alternated with 30 s of rest, on ice. The lysate was centrifuged at 8000 × g for 10 min to isolate the cytoplasmic soluble protein. The pellet was recovered in 0.1% of Nonidet-P40, and incubated at 4°C for 2 h and centrifuged at 8000 × g for 10 min. Then, the pellet was suspended in PBS and 3 μL of MgSO_4_ (1 M) were added, and it was submitted to DNase I treatment for 3 h. Thereafter, IBs were recovered by centrifugation and the pellet was washed with 0.5% Triton X-100 for 2 h at 4°C. Then the pellet was washed twice with deionized water to remove the excess of salts and detergent. The solution was centrifuged for 30 min at 8000 × g and the solids obtained were washed 3 times with deionized low conductivity water. Finally, the IB were stored at -80°C [10],[101],[102].

The concentration of total, cytoplasmic soluble and IB proteins was determined by 2D-Quant kit (G-Biosciences, USA), following the supplier recommendations. IBs were suspended in denaturing buffer (Tris-HCl 250 MM pH 6.8, 40% v/v glycerol and 5% v/v SDS) [103] to measure protein concentration, and incubated at 24-27°C for 12 h in order to obtain a complete dissolution of the aggregates. Calibration curves were prepared using BSA. Samples and standards were prepared at least by duplicate and measured at 480 nm in a plate reader.

### Sphingomyelinase-D protein identification and qualitative measurement activity

The recombinant sphingomyelinase-D (rSMD) expression was confirmed by SDS-PAGE [94] and Western Blot. Samples were collected at different times to analyze the soluble protein as well the recombinant protein in the IB. The 15% SDS gels were stained with Coomassie Brilliant Blue R-250, and quantification was done by densitometry using the Image-Lab™ software and Gel Doc™ EZ System (Bio-Rad, USA). For Western Blot, the cytoplasmic soluble proteins and the proteins solubilized from IBs were separated on 15% SDS-PAGE under reducing conditions. Then, they were transferred to a polyvinylidene difluoride (PVDF) membrane (Millipore, USA), which was blocked in buffer PBS, Tween-20 (0.5%), and BSA (3%). Incubated with 1:5000 mouse Anti-His_6_-Peroxidase antibody IgG_1_ (Roche, USA) for 60 min at 25°C. The immunoreactive bands were detected by chemiluminescence using SuperSignal West Pico Chemiluminescent Substrate (Thermo Scientific, USA) and visualized using the C-DIGIT blot scanner (LI-COR, USA). Qualitative SMD activity from soluble and IB proteins, was confirmed coupling the assay using the substrate AMPLEX (Molecular Probes) and fluorimetric detection as described by the manufacturer and reported by Ramos-Cerrillo et al. [77]. Sphingomyelinase C from *Staphylococcus aureus* was used as positive control and reference standard. The negative control consisted in the same reaction without protein sample.

### Size analysis of IBs inside cells

Morphology and size were analyzed under transmission electron microscopy. Cell samples and IBs were taken at different kinetics times, washed three times with 0.16 M sodium cacodylate buffer at pH 7.2 at 4°C, fixed with 4% paraformaldehyde and 2.5% glutaraldehyde in sodium cacodylate buffer pH 7.4 during 2 h at 4°C. Post-fixed samples with 1% osmium tetraoxide during 90 min at 4°C, were rinsed twice in chilled buffer and six times in cold distilled water. Then, samples were dehydrated in ethanol series and embedded in Epon/Araldita [104]. Thin sections were stained with uranyl acetate and lead in citrate and observed with a ZEISS Libra 120 plus electron microscope. At least 100 cells were analyzed for each sample, and samples were obtained from two independent cultures for each pH strategy.

### Size analysis of inclusion bodies by dynamic light scattering

The hydrodynamic diameter of the IBs harvested at different time points after induction was determined by dynamic light scattering (DLS) performed in a Zetasizer Nano ZS (Malven Inst. Ltd, Worcestershire, UK) at 173° backscatter using a 50-μL quartz cuvette [105]. Samples were analyzed with and without centrifugation to evaluate the size and quality of the IBs by using the normal resolution mode. Absorbance values at 350 nm were acquired and samples were diluted with water to obtain an absorbance value of 0.5 AU before carrying out the measurement by DLS. Sizes are reported as the diameter of the equivalent sphere of the particles analyzed [105]. DLS has been recently widely used to determine the hydrodynamic diameter of IBs [12],[23],[25],[61],[106]-[108]. Each sample was measured in triplicate and the hydrodynamic diameter represents the mean value. Dispersants used in this manuscript were water and PBS buffer. All samples were analyzed at 27°C. To evaluate the IB stability in the solvents used, the scattering intensity and hydrodynamic diameter were monitored as a function of time.

The IB_50_ was defined as the maximum size reached by 50% of the IB population, and can be seen as the statistical median equal to the 50th percentile. Also, the arithmetic mean (average) was calculated for all collected samples. In order to measure the broadness and the deviation of the log-normal distribution of IB sizes we defined the arithmetic mean/IB_50_ ratio as a form to compare the polydispersity of the IB sizes.

### Proteolytic digestion of inclusion bodies

The IBs containing rSMD harvested from two different pH culture strategies after induction were digested using proteinase-K at 12 μg/mL (final concentration). The proteolytic digestion was carried out with 150 μg/mL of IBs suspended in 50 MM Tris-HCl, 150 MM NaCl pH 8.0 buffer, and it was monitored for 100 minutes measuring the changes in optical density at 350 nm in UV-2450 spectrophotometer (DU 730 Beckman coulter USA) [61].

### Solubilization of purified IBs

The solubilization profile of purified IBs was determined using different concentrations of guanidine hydrochloride (GnCl). The IBs were solubilized in the presence of different concentrations of GnCl (0 M to 5 M), and 50 MM Tris-HCl, 5 MM DTT, pH 8.5, during 24 h [31],[32]. The initial concentration of proteins in the IBs was in the range of 0.1 Mg/ml. The IB solubilization was determined measuring the liberation of protein by 2D-Quant kit (G-Biosciences, USA), following the supplier recommendations.

### Amyloid specific assays of IB using dyes

A spectroscopy assay was used to determine the beta sheet conformation inside the IB by using Congo red and analyzing the band shift [9],[61]. A total of 50 μg of protein in IBs was diluted in buffer (10 MM sodium phosphate, pH 7.0 containing 150 MM NaCl) with 10 μm of Congo red. Each sample was incubated for 10 min at room temperature before the spectra acquisition data in UV-2450 spectrophotometer (DU 730 Beckman coulter USA). The change in absorbance spectra was measured in the wavelength range of 400 to 700 nm. The spectra controls were obtained from the dye in the absence of protein, and form protein in absence of dye, which were subtracted from their respective problem samples [61]. The fluorescence from the binding of Thioflavin-T to IB was analyzed using a spectrofluorometer Luminescence spectrometer LS55 (Perkin Elmer Instruments, MA; USA). 50 μg/mL of IBs were diluted in 10 MM phosphate pH 7.0 buffer, 150 MM NaCl, and 75 MM Thioflavin-T [61]. The reaction product was incubated for 1 h at room temperature. The emission spectra were recorded from 460 to 600 nm using an excitation wavelength of 440 nm [61]. Slit widths of 5 nm were used for both excitation and emission, and a scan speed rate of 50 nm/min. The data were acquired with the FLWinlab software (Perkin Elmer Instruments, MA; USA).

## Authors’ original submitted files for images

Below are the links to the authors’ original submitted files for images. Authors’ original file for figure 1 Authors’ original file for figure 2 Authors’ original file for figure 3 Authors’ original file for figure 4 Authors’ original file for figure 5 Authors’ original file for figure 6 Authors’ original file for figure 7 Authors’ original file for figure 8
