# Supplementary figures and images for: Influence of pH control in the formation of inclusion bodies during production of recombinant sphingomyelinase-D in Escherichia coli
Source: Microb Cell Fact. 2014 Sep 12;13:137. doi: 10.1186/s12934-014-0137-9 (PMC4177172; doi:10.1186/s12934-014-0137-9)

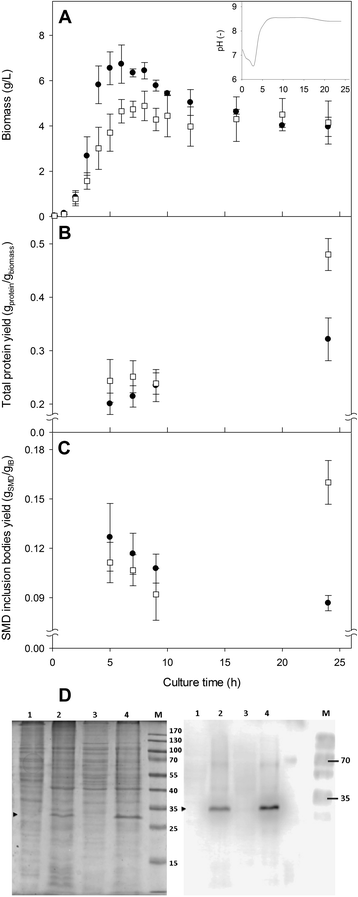

Supplement: Supplementary file 1 — Authors’ original file for figure 1 [file 12934_2014_137_MOESM1_ESM.gif]

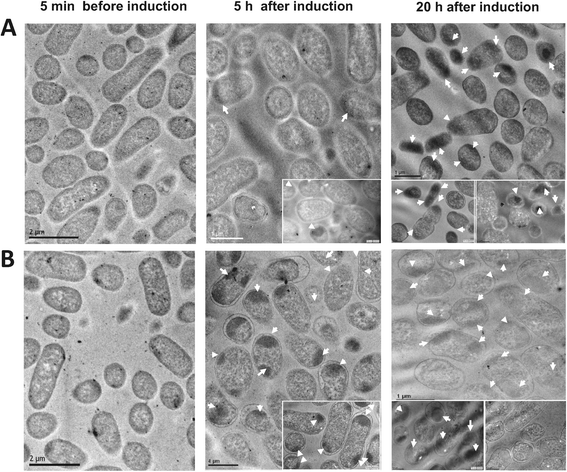

Supplement: Supplementary file 2 — Authors’ original file for figure 2 [file 12934_2014_137_MOESM2_ESM.gif]

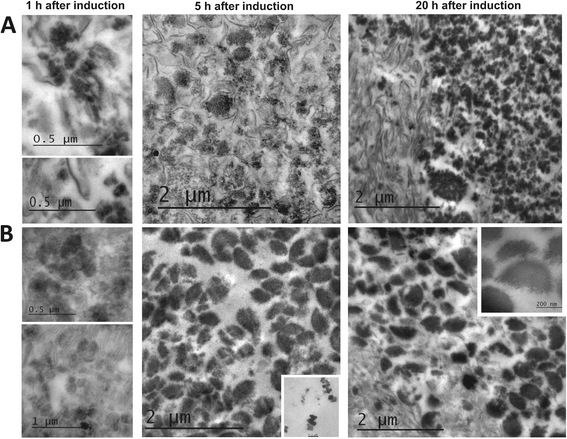

Supplement: Supplementary file 3 — Authors’ original file for figure 3 [file 12934_2014_137_MOESM3_ESM.gif]

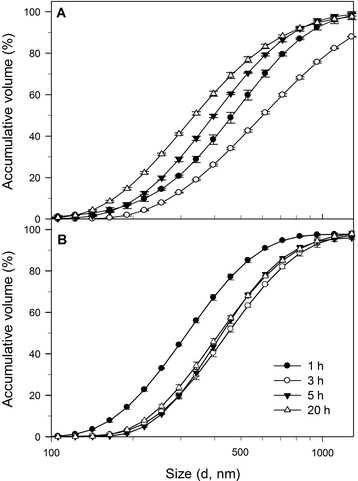

Supplement: Supplementary file 4 — Authors’ original file for figure 4 [file 12934_2014_137_MOESM4_ESM.gif]

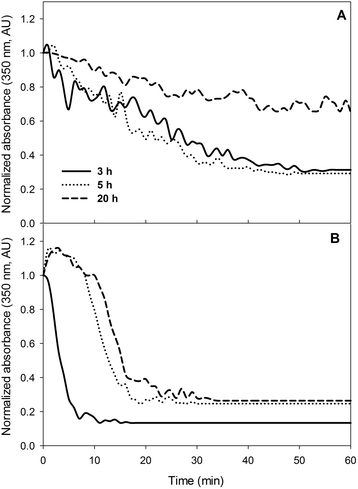

Supplement: Supplementary file 5 — Authors’ original file for figure 5 [file 12934_2014_137_MOESM5_ESM.gif]

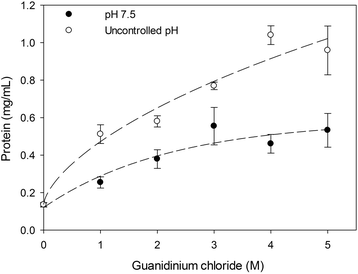

Supplement: Supplementary file 6 — Authors’ original file for figure 6 [file 12934_2014_137_MOESM6_ESM.gif]

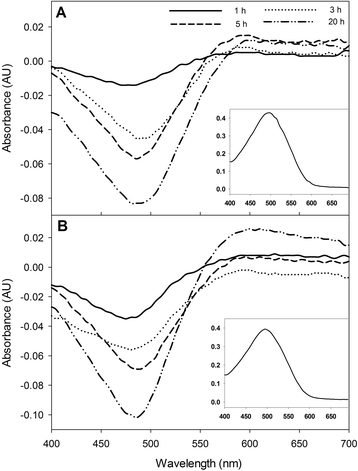

Supplement: Supplementary file 7 — Authors’ original file for figure 7 [file 12934_2014_137_MOESM7_ESM.gif]

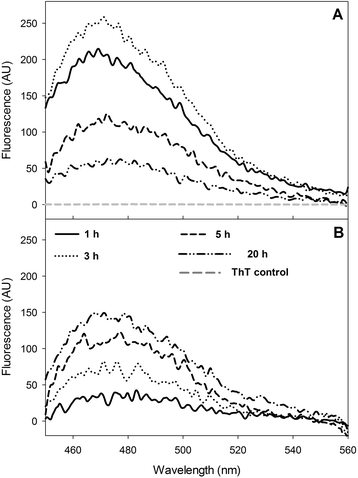

Supplement: Supplementary file 8 — Authors’ original file for figure 8 [file 12934_2014_137_MOESM8_ESM.gif]
